# Integrating Multi-Omics and Medical Imaging in Artificial Intelligence-Based Cancer Research: An Umbrella Review of Fusion Strategies and Applications

**DOI:** 10.3390/cancers17223638

**Published:** 2025-11-13

**Authors:** Ahmed Al Marouf, Jon George Rokne, Reda Alhajj

**Affiliations:** 1 Department of Computer Science, University of Calgary, Calgary, AB T2N 1N4, Canada; rokne@ucalgary.ca; 2 Department of Computer Engineering, Istanbul Medipol University, Istanbul 34810, Turkey; 3 Department of Health Informatics, University of Southern Denmark, 5230 Odense, Denmark

**Keywords:** multi-omics, medical imaging, cancer, artificial intelligence, umbrella review, fusion strategies

## Abstract

**Background:** The combination of multi-omics data, including genomics, transcriptomics, and epigenomics, with medical imaging modalities (PET, CT, MRI, histopathology) has emerged in recent years as a promising direction for the advancement of precision oncology. Many researchers have contributed to this domain, exploring the multi-modality aspect of using both multi-omics and image data for better cancer identification, subtype classifications, cancer prognosis, etc. **Methods:** We present an umbrella review summarizing the state of the art in fusing imaging modalities with omics and artificial intelligence, focusing on existing reviews and meta-analyses. The analysis highlights early, late, and hybrid fusion strategies and their advantages and disadvantages, mainly in tumor classification, prognosis, and treatment prediction. We searched review articles until 25 May 2025 across multiple databases following PRISMA guidelines, with registration on PROSPERO (CRD420251062147). **Results:** After identifying 56 articles from different databases (i.e., PubMed, Scopus, Web of Science and Dimensions.ai), 35 articles were screened out based on the inclusion and exclusion criteria, keeping 21 studies for the umbrella review. **Discussion:** We investigated prominent fusion techniques in various contexts of cancer types and the role of machine learning in model performance enhancement. We address the problems of model generalizability versus interpretability within the clinical context and argue how these multi-modal issues can facilitate translating research into actual clinical scenarios. **Conclusions:** Lastly, we recommend future work to define clearer and more reliable validation criteria, address the need for integration of human clinicians with the AI system, and describe the trust issue with AI in cancer care, which requires more standardized approaches.

## Simple Summary

This study reviews how combining different types of biological data (like genes and proteins) with medical images (such as MRI, CT, or PET scans) can help improve cancer diagnosis and treatment. The authors looked at many review papers published before May 2025 and selected 21 that best fit their goals. The review explains how researchers are mixing these data types to better detect cancer, predict patient outcomes, and guide treatment choices. It also discusses common challenges, such as how to make these AI-based systems both accurate and understandable for doctors. The paper ends by stressing the importance of building more trustworthy and human-centered AI systems for use in real medical settings.

## 1. Introduction

Medical imaging techniques such as Positron Emission Tomography (PET), Computed Tomography (CT), Magnetic Resonance Imaging (MRI) and histopathological imaging have become indispensable tools in modern clinical practice and biomedical research. These imaging modalities provide complementary information at various spatial and functional levels, supporting disease diagnosis, staging, and evaluation of response to treatment. PET imaging is widely used for functional imaging by capturing metabolic activity, while CT and MRI offer high-resolution anatomical and soft tissue contrast, respectively [1]. Histopathological images, derived from biopsy specimens, allow microscopic examination of tissue morphology, serving as the gold standard for cancer diagnosis and grading [2]. Despite their power, these imaging techniques traditionally operate in silos, often failing to fully capture the biological complexity of diseases such as cancer.

In parallel, advances in high-throughput technologies have enabled complete profiling of the molecular landscape of diseases through multi-omics approaches, encompassing genomics, transcriptomics, epigenomics, proteomics, and metabolomics. Large-scale initiatives such as the Cancer Genome Atlas (TCGA) and the Genotype-Tissue Expression (GTEx) project have facilitated the integration of these diverse data types into data sets containing thousands of multiomics patient samples [3]. This data provide granular insight into gene regulation, mutational landscapes, epigenetic modifications, and cellular signaling pathways, offering the potential for highly personalized medicine. Interpreting and integrating such heterogeneous and high-dimensional data poses significant analytical challenges however, often requiring sophisticated computational methods.

The convergence of medical imaging and multiomics data has led to the development of multimodal artificial intelligence (AI) approaches that aim to leverage the complementary strengths of each modality for enhanced disease characterization and prediction. This has been validated in recent studies that have demonstrated the value of integrating radiological or histopathological images with omics data for an improved cancer prognosis, treatment stratification, and biomarker discovery [4,5]. These multimodal models often employ deep learning architectures, such as convolutional neural networks (CNNs) and transformers, alongside graph-based or attention-based fusion strategies to capture complex interdependencies between modalities [6]. As AI-driven multimodal frameworks continue to evolve, they hold immense promise for advancing precision medicine as well as for uncovering novel insights into disease mechanisms.

### 1.1. Multimodal Data in Oncology

The emergence of multimodal data, particularly the integration of medical imaging and multiomics, has revolutionized cancer research and clinical oncology by enabling a more comprehensive understanding of tumor biology. Medical imaging modalities such as CT, MRI, PET, and histopathology provide spatial and structural context, while multiomics data, including genomics, transcriptomics, proteomics, and epigenomics, offer molecular-level insights into tumor heterogeneity and evolution. The complementary nature of these data types allows for a more holistic analysis of cancer phenotypes, linking morphological features with underlying biological mechanisms. For example, radiogenomic studies have demonstrated correlations between imaging characteristics and gene expression profiles, suggesting that noninvasive imaging can serve as a proxy for molecular characterization [7,8].

Advances in artificial intelligence (AI) and deep learning have further empowered the fusion of these multimodal data sets to develop predictive models for diagnosis, prognosis, and treatment response. Integrated frameworks that combine histopathological images with genomic profiles have shown improved performance in predicting patient outcomes and identifying molecular subtypes compared to unimodal approaches [9]. Deep learning architectures such as convolutional neural networks (CNNs), attention mechanisms, and multimodal fusion models (e.g., transformers and graph-based networks) are increasingly being used to manage the high dimensionality and heterogeneity of these datasets [10,11]. As large-scale initiatives such as The Cancer Genome Atlas (TCGA) (https://www.cancer.gov/ccg/research/genome-sequencing/tcga, accessed on 1 October 2025) and The Cancer Imaging Archive (TCIA) (https://www.cancerimagingarchive.net, accessed on 1 October 2025) continue to grow, multimodal approaches are expected to play a crucial role in precision oncology, guiding clinical decision making with greater precision and interpretability.

### 1.2. The Need for AI-Based Fusion

The integration of multi-omics and medical imaging data presents a unique opportunity for comprehensive cancer characterization, but the inherent heterogeneity and high-dimensionality of these modalities require advanced computational fusion techniques. Traditional statistical fusion methods struggle to capture complex non-linear relationships between molecular and imaging characteristics, prompting the adoption of AI-based fusion strategies. Information fusion in this context can be broadly categorized into early fusion (feature level), late fusion (decision level) and hybrid fusion. Early fusion involves concatenating raw or preprocessed features from each modality before feeding them into a unified model, enabling the model to learn joint representations across data types. This strategy is particularly useful when the modalities are temporally or spatially aligned, as it allows deeper cross-modal interactions [4,12]. However, early fusion may suffer from overfitting because of the high dimensionality and lack of modality-specific preprocessing.

This is in contrast to late fusion, which processes each modality independently through dedicated networks or pipelines and combines the output at the decision level. This modularity allows each sub-network to specialize in a particular data type, improving interpretability and model robustness, especially when modalities vary in data quality or availability.

Hybrid fusion strategies attempt to capitalize on the strengths of both approaches by combining features at multiple levels, for example, integrating early fusion representations with decision-level fusion outputs, to enhance predictive accuracy and biological relevance. Recent studies have shown that hybrid architectures, including attention mechanisms and graph neural networks, can effectively model complex inter-modal relationships in cancer prognosis and prediction of response to treatment [10,13,14].

As the volume and complexity of biomedical data continue to grow, the development of scalable and interpretable fusion frameworks will be critical to realize the full potential of multimodal precision oncology.

### 1.3. The Need for an Umbrella Review

Despite the rapid expansion of research at the intersection of multiomics, medical imaging, and artificial intelligence (AI) in oncology, the field remains fragmented with diverse methodologies, inconsistent reporting standards, and varying levels of evidence. Existing reviews typically focus on individual modalities or specific types of cancer, limiting the ability to generalize findings or identify robust fusion strategies. An umbrella review—synthesizing evidence in multiple systematic reviews and meta-analyses—can provide a comprehensive, high-level understanding of the field, highlighting current integration practices, model architectures, evaluation metrics, and clinical applicability [15]. This review is therefore critical to identify methodological gaps, compare fusion strategies (early, late and hybrid), and propose standardized frameworks for reproducibility and interpretability in multimodal AI-driven oncology research. In addition, it can guide future studies by synthesizing evidence on the most effective combinations of imaging and omics data for different clinical endpoints. As there is currently no umbrella review in this niche, its development is timely and necessary in order to consolidate fragmented knowledge and accelerate translational impact as noted by [16].

The key contribution of this umbrella review lies in the synthesizing of multimodal fusion strategies across both omics and imaging datasets—something not addressed collectively by existing reviews. This study uniquely maps (i) fusion architectures, (ii) multimodal data availability, (iii) evaluation practices, and (iv) clinical interpretability, providing a translational research perspective for precision oncology.

## 2. Materials and Methods

### 2.1. Study Protocol

**Figure 1:**
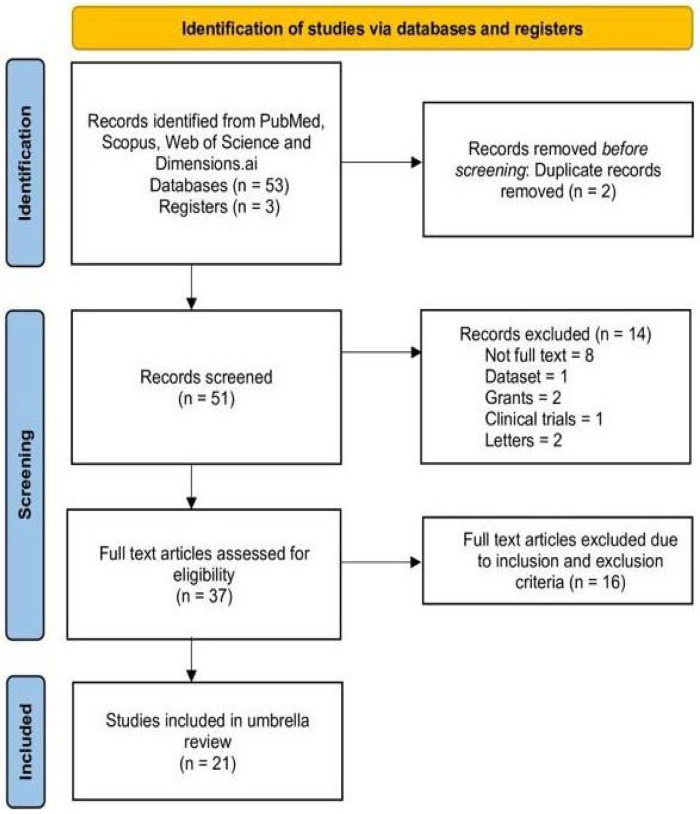
Flow diagram for study selection process using PRISMA 2020 model.

For the umbrella review study, we have adopted the Preferred Reporting Items for Systematic Reviews and Meta-Analyses (PRISMA) model [17,18]. The step by step flow diagram of PRISMA has been depicted in Figure 1. The study has been registered on the Prospective Register of Systematic Reviews (PROSPERO) [19], being made publicly available under the registration number CRD420251062147, published on 29 May 2025.

### 2.2. Leveraging PICOS Framework

The widely used PICOS (Population, Intervention, Comparison, Outcomes and Study) framework [20,21] was used for the umbrella review. The PICOS elements for this study have been presented in Table 1. We considered only cancer patients whose data have been utilized by AI models in different studies. The study intervened with AI-based fusion techniques using multi-omics and medical imaging processes. There was no particular comparator and different results were considered, such as precision for prediction or classification methods, interpretability for interpretable AI or explainable AI methods, and clinical values for clinical trails. In addition, systematic reviews and meta-analyzes of the literature performed by researchers are included. The search strategy, inclusion, and exclusion criteria define the final list of reviews included in this study.

### 2.3. Search Strategy

For this study, we searched four main research databases that have been used for similar review studies. The databases we searched are PubMed (https://pubmed.ncbi.nlm.nih.gov/, accessed on 1 October 2025), Scopus (https://www.scopus.com/home.url, accessed on 1 October 2025), Web of Science (WoS) (https://www.webofscience.com/wos/woscc/advanced-search, accessed on 1 October 2025), and Dimensions.ai (https://www.dimensions.ai/, accessed on 1 October 2025). We conducted this systematic search on these four databases from their inception to 25 May 2025. The keywords used for database searches are listed in Table 2. The comprehensive search strategy was applied using Boolean expression across multiple biomedical concepts (systematic reviews, multi-omics, imaging, cancer, fusion, and AI). For searches, the available advanced search technique tools from the databases were used.

### 2.4. Inclusion & Exclusion Criteria

The inclusion and exclusion criteria for this broad review on AI/ML-based fusion of omics and imaging in cancer research were established as shown in Table 3. To be eligible for inclusion in the study, the article had to be a review or meta-analysis. There might be several domains or focus points in the review articles, but only the articles with a specific focus on AI/ML-based fusion of different omics and medical imaging data have been included. Moreover, studies had to focus on cancer research. Original research articles were excluded. Reviews included in the study had to discuss artificial intelligence, machine learning, or deep learning-related fusion using omics and/or imaging data. Articles focusing only on either omics reviews or image modality reviews were also included since they might lead to possible fusion approaches. However, reviews focusing on specific topics, such as a particular gene signature, were excluded. Moreover, studies that involved non-human models or non-cancer conditions were also excluded in the screening process.

### 2.5. Data Extraction

To ensure systematic and collaborative data extraction, we employ a structured workflow using Rayyan (https://www.rayyan.ai/, accessed on 1 October 2025), an online platform that allows simultaneous screening by multiple reviewers to finalize the selection of included articles. A standardized shared spreadsheet was generated to extract key data fields from each study, including authors, publication year, cancer type studied, multi-omics (e.g., genomics, proteomics) and imaging modalities (e.g., MRI, histopathology), data fusion approaches (e.g., early/late fusion), AI methods (e.g., deep learning, ensemble models), study tasks (e.g., diagnosis, prognosis), and main outcomes. Two reviewers independently performed manual extraction, and discrepancies were resolved by consensus or adjudication by a third reviewer. Data extracted from 18 publications were consolidated into a comprehensive table (Table 4) for comparative analysis. To enhance the rigor of selection of articles, we followed predefined extraction criteria and maintained transparency throughout the process, although future improvements could include pilot testing of the extraction form and the integration of automated tools for efficiency.

### 2.6. Quality Assessment Using AMSTAR 2.0

Achieving an umbrella review of high quality is extremely important as we aim to take advantage of the review in the future in several research domains. Keeping this in mind, we have adopted the widely accepted AMSTAR 2.0 version [22] for our umbrella review study. AMSTAR is an acronym for A MeaSurement Tool to Assess systematic Reviews. Although the main idea for the article presented by Beverley J. Shea et al. in [23] in 2009 and [22] in 2017 was generally applicable for the quality assessment of systematic reviews, the researchers have adopted the same assessment for scoping reviews, meta-analysis, and umbrella reviews [24,25].

AMSTAR 2.0, consisting of a 16-item checklist, which is provided at https://amstar.ca/Amstar_Checklist.php (accessedon 1 October 2025), has been used to verify that all quality assessment requirements are maintained appropriately. This umbrella review fully adheres to AMSTAR 2.0 standards following the checklist, ensuring rigorous methodology and transparency. The PICO framework was explicitly defined, focusing on multi-omics and imaging fusion (genomics, transcriptomics, PET, MRI, etc.) for cancer classification, prognosis, and treatment response prediction. A preregistered protocol (PROSPERO CRD420251062147) guided the review process, aligning it with the PRISMA reporting guidelines.

A comprehensive literature search was conducted on PubMed, Scopus, Web of Science, and Dimensions.ai, with dual independent screening and data extraction performed by AAM and JGR to minimize bias. The risk of bias in included reviews was assessed using the ROBIS tool [26], and the results were documented in the Supplementary Materials. Publication bias was evaluated using funnel plots (presented in the Results section), and primary study overlap was quantified using Pieper’s matrix, confirming minimal redundancy. The review systematically compared early, late, and hybrid fusion techniques, addressing heterogeneity through subgroup analysis. Conflict of interest statements were included, with no financial or professional biases identified. By integrating dual reviewer validation, bias assessments, and overlap analyzes, this study achieves a high level of confidence. A discussion section highlights the clinical translation challenges (e.g., interpretability, generalizability) while proposing standardized validation criteria for future AI-driven multimodal oncology research.

### 2.7. Data Synthesis

We looked at the data and explained the findings in words, as well as in tables and charts. Because the studies we included have some heterogeneity, we could not combine their results into one general analysis. Instead, we compare our findings with what other research has shown, and we point out both the strengths and the weaknesses of our review.

## 3. Results

This section provides a detailed overview of the articles that are most relevant to the topic of our umbrella review. We have included details of the risk of bias selection characteristics and key findings of the included study in this umbrella review.

### 3.1. Characteristics of Included Reviews

Using the keywords presented in Table 2 and following the PRISMA guidelines shown in Figure 1, we searched PubMed, Scopus, Web of Science and Dimensions.ai. The summary of the search results are provided in Table 4.

We found 53 articles with two duplicate records and three registers. After applying dual screening, 51 articles were found in the initial stage. After excluding non-full texts, datasets, grants, clinical trials, and letters, 37 articles were considered eligible for full-text reading. Following inclusion and exclusion criteria, 21 systematic reviews were finally included for qualitative and quantitative synthesis.

### 3.2. Summary of Umbrella Review

In this section, we summarize the 21 selected studies. The publication year, cancer types, omics and image modalities used for the study, fusion type implemented, and AI methods used. The selected studies and their summarization, which is the main result of the study, are presented in Table 5 and Table 6. The main contributions and limitations of the studies are also presented in Table 6. To simplify further processing or quality measurement via AMSTAR 2.0, we have assigned identifiers (ID) in the Tables. We used the same IDs for further discussion and plots.

### 3.3. Methodological Quality Assessment Using AMSTAR 2.0

We used the AMSTAR 2.0 criteria to measure the quality of the 21 review articles (shown in Table 6) selected for this umbrella review. Unlike PROBAST, a tool for assessing the risk of bias and applicability of prediction models [48,49], there is no specific scoring to interpret the quality of a review by the AMSTAR 2.0 system. The level of measurements used in AMSTAR 2.0 are low, moderate and critically low. Only 1 of 21 (4.8%) studies was classified as moderate. 7 out of 21 (33.3%) were classified as low, and the remaining 13 (61.9%) articles were classified as critically low. The results of the evidence measurement are presented in Table 7. We used the abbreviations N, NR, Y, PN and NA in the table to assess each study. In the AMSTAR 2 assessment, the domains were rated as follows: Y (Yes) when the criterion was clearly satisfied (e.g., the study reported a registered protocol or used a comprehensive search strategy); PN (Partial/Unclear) when the criterion was only partially fulfilled or the reporting was ambiguous (e.g., limited database search without full detail); N (No) when the criterion was not satisfied (e.g., absence of protocol registration or risk of bias assessment); NR (Not Reported) when the item was not mentioned at all in the study; and NA (Not Applicable) when the item was not relevant to the type of review (e.g., meta-analytic methods in narrative reviews without quantitative synthesis).

**Figure 2:**
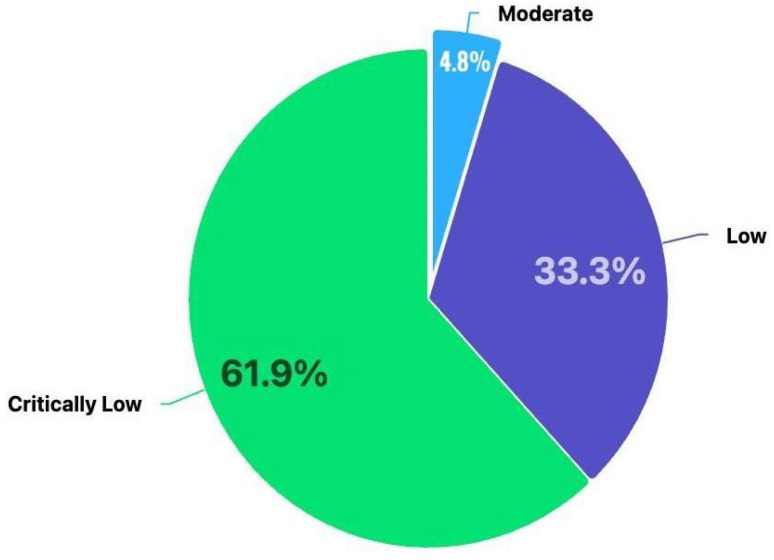
Distribution of AMSTAR 2 methodological quality ratings across 21 included studies.

The summarization using these quality assessment conditions is shown in Figure 2.

### 3.4. Fusion Strategies Findings and Criticisms

The integration of multi-omics and imaging data in cancer research is commonly framed around early, late, and hybrid fusion approaches. Early fusion strategies typically combine raw or pre-processed features from different modalities into a single input for a machine learning model. This approach has been widely reviewed in the context of multiomics integration, where genomic, transcriptomic, and epigenomic profiles are concatenated to capture complementary signals for cancer classification and prediction of subtypes [27,28,33,37]. Although early fusion offers the advantage of exploiting the joint distribution of heterogeneous features, it often suffers from the curse of dimensionality and thus demands careful preprocessing and normalization across data types [32,35].

In contrast, late-fusion strategies process each modality independently and combine the outputs at a decision level. For example, separate models can be trained on radiomic features, histopathology images, and omics data, with their predictions aggregated through ensemble methods or meta-learners [29,39,40]. This approach provides greater flexibility in handling heterogeneous data and allows each model to specialize in its modality. Reviews in cancer imaging have therefore emphasized the utility of late fusion, especially in radiomic and PET-based umbrella analyzes, where modality-specific predictors are integrated for diagnostic precision or prediction of treatment response [36,40]. However, late fusion may not capture subtle interactions across modalities that are only evident when data are combined at the feature level.

To address these limitations, hybrid fusion strategies that combine aspects of early and late integration have emerged. Hybrid models often extract intermediate latent representations from each modality before merging them in a joint feature space, which can then be used for downstream tasks such as prognosis, biomarker discovery, or prediction of therapy response [30,31,41,44]. Several systematic reviews report that hybrid strategies outperform unimodal and purely early or late fusion in terms of predictive performance and robustness [39,43,45]. For example, biologically informed deep learning frameworks highlight the potential to integrate domain knowledge into hybrid models, improving interpretability while retaining accuracy [31,47].

In general, the evidence from the selected reviews suggests that fusion strategies are evolving toward more context-aware and knowledge-driven integration frameworks. Hybrid approaches, particularly those informed by biological priors or clinical constraints, appear to provide a balance between performance and interpretability [44,47]. However, challenges remain in standardizing fusion pipelines, defining optimal integration levels for different types of cancer, and ensuring reproducibility across diverse datasets [34,38,46]. Future work is likely to focus on adaptive, task-specific fusion methods that can dynamically weight modalities based on the clinical context, thus moving closer to the goal of reliable precision oncology.

### 3.5. Publicly Available Clinical and Multi-Omics Imaging Datasets

The rapid evolution of multimodal AI in oncology has been driven by the availability of large-scale public datasets that combine molecular profiling with clinical and imaging data. These repositories enable reproducible research, comparative benchmarking, and accelerate model development for the diagnosis, prognosis, and therapeutic stratification of cancer. Table 8 summarizes the key datasets commonly used in multimodal fusion research, highlighting the modality types (e.g., PET/CT/MRI, histopathology slides), the omics layers (e.g., genomics, transcriptomics, proteomics), the coverage of cancer, and access status.

The Cancer Genome Atlas (TCGA) [50] and The Cancer Imaging Archive (TCIA) [51] are the most widely used paired repositories, supporting the direct integration of genomic and imaging modalities. CPTAC [52] expands this by incorporating proteomics, enabling in-depth proteogenomic research. UK Biobank [53] offers population-scale imaging related to genomics and clinical phenotypes, suitable for longitudinal and early-risk prediction studies. SEER [54] provides demographic and survival statistics for population-level outcome modeling. Finally, multi-institutional radiomics datasets [55]—such as LIDC-IDRI and NSCLC-Radiomics—enable standardized benchmarking of segmentation and radiomics models.

Together, these datasets represent the foundational resources for multimodal AI in cancer. Their structured data availability supports methodological transparency, facilitates external validation, and drives clinical translation of fusion-based AI systems.

## 4. Discussion

### 4.1. Insights Across Cancer Types

The reviewed studies demonstrate that the integration of multiomics and imaging modalities has been applied across a wide spectrum of cancer types, with heterogeneous levels of maturity. For gastrointestinal cancers, multiomics-based diagnosis surveys indicate that combining transcriptomic, genomic, and epigenomic characteristics provides a significant increase in classification accuracy compared to single-omics models [27]. In ovarian cancer, systematic reviews highlight the value of combining genomics, radiology, and immunotherapy biomarkers, reporting improved performance for prognosis and prediction of therapy response [41]. Renal cell carcinoma studies illustrate how AI-enhanced fusion can assist in diagnostic challenges by combining radiological features with molecular data [43]. Glioblastoma has been a key focus for imaging–omics integration, particularly in predicting the methylation status of MGMT from MRI along with epigenomic data, offering non-invasive biomarkers for clinical decision making [40,45]. In addition, umbrella reviews in PET imaging confirm that decision-level fusion of radiopharmaceutical data between cancer types can guide tracer selection and treatment planning [36]. Collectively, these insights indicate that while certain cancers such as glioblastoma, ovarian, and renal malignancies are well represented, others like breast and prostate cancers remain underexplored in the context of multi-omics and imaging integration.

### 4.2. Gaps & Challenges

Despite promising advances, several methodological and practical gaps remain evident across these reviews. A critical limitation is the lack of standardized pipelines for multi-omics and imaging fusion, leading to inconsistent reporting and limited reproducibility [28,32,44]. Many reviews emphasize that the heterogeneity of data sources, pre-processing methods, and feature extraction techniques introduces substantial variability in model performance [34,38,46]. Another gap lies in the insufficient consideration of bias and generalizability: most studies rely heavily on retrospective cohorts such as TCGA, with few employing prospective validation or multi-center datasets [33,35,42]. Moreover, risk of bias assessments are rarely performed, contributing to the prevalence of critically low methodological quality in these reviews [27,30,31]. There is also an underutilization of biologically informed or knowledge-constrained AI methods, which could bridge the gap between model accuracy and interpretability [31,47]. Finally, the computational demands of high-dimensional data integration remains a barrier to scalability, particularly in resource-limited settings [45].

### 4.3. Crosstalk Between AI and Clinical Base

A recurring theme in the reviews is the challenge of translating AI-driven fusion models into clinically actionable tools. Although hybrid fusion strategies consistently demonstrate superior performance, their complexity raises concerns regarding interpretability and integration into existing clinical workflows [39,41,44]. Reviews on personalized PET imaging highlight how umbrella-level evidence can inform tracer selection, suggesting a direct path toward clinical decision support [36]. Similarly, glioblastoma MGMT prediction studies exemplify how imaging–omics models could be used in practice to reduce invasive biopsies [40,45]. However, most reviews caution that trust, validation, and patient participation remain essential to clinical uptake [35,43,47]. The literature calls for cross-talk between AI developers and clinicians, emphasizing the co-design of fusion frameworks that align with clinical guidelines, reporting standards, and workflow constraints [37,41]. Future translation will depend not only on algorithmic accuracy but also on regulatory readiness, cost-effectiveness evaluations, and integration of multimodal models into multidisciplinary tumor boards. This indicates a strong need for the development of consensus-driven validation protocols as well as closer partnerships between computational scientists and healthcare providers to bridge the gap.

### 4.4. Evaluation Metrics Used in Multi-Modal Fusion Studies

Evaluation metrics differ across studies in multimodal cancer research depending on the prediction task and data modality. For classification problems, such as subtype detection or recurrence prediction, the most commonly used measures are AUC-ROC and F1-score. AUC-ROC is preferred in clinical settings because it remains robust in class imbalance, a frequent issue in rare cancer datasets [56]. The F1-score provides a balance between precision and recall, complementing AUC-ROC, especially when false negatives need to be minimized. The Concordance Index, or C-index, is the standard metric for survival prediction and prognostic modeling using multi-omics data, which captures the quality of the ranking of predicted survival times [57]. For segmentation tasks, which often form a step preceding feature extraction in radiomics or histopathology, metrics such as Dice Similarity Coefficient (DSC) quantify overlap between predicted and ground truth anatomical regions [58]. Metric selection can vary greatly depending on the task at hand across the range of multimodal fusion studies, and thereby, a rigorous evaluation of several metrics for translational validity is encouraged.

### 4.5. Explainable Artificial Intelligence (XAI) in Multimodal Cancer Modeling

It is explainability that will allow the translation of multimodal AI into clinical oncology; clinicians want to understand how imaging and omics features drive model outputs. Approaches to XAI span four broad classes: saliency-based, attribution-based, attention-based, and counterfactual methods. Saliency methods such as Grad-CAM highlight areas in PET/CT or histopathology images relevant to decision-making [59]. Exciting new counterfactual explanations will enable clinicians to ask “what needs to change in the input (levels of a biomarker or shape of a tumor) to change the prediction?” [60]. XAI bridges statistical prediction with mechanistic interpretation within multimodal cancer modeling, reducing trust gaps and enhancing acceptance in decision-support workflows.

Abbas et al. [61] in their meta-analysis review the current landscape of explainable artificial intelligence (XAI) within clinical decision support systems (CDSSs), synthesizing how different methods (e.g., SHAP [62], LIME, attention maps) are applied across healthcare domains. It identifies key usability challenges, including the absence of longitudinal clinical validation, limited evaluation of explanation fidelity, and sparse reporting of clinician trust or workflow integration outcomes. The authors argue for a stronger focus on participatory system design, consistent interpretability reporting, and the development of domain-specific XAI frameworks to help bridge the gap between high performance AI systems and real-world clinical adoption [61].

In the domain of high-stakes decision-making such as precision oncology, model transparency and interpretability are indispensable for building clinician trust and enabling meaningful oversight. Mookkandi & Nath et al. [63] proposed a hybrid deep neural network combining channel-attention and inception-residual modules for crop-disease classification, highlighting the importance of attention mechanisms to isolate salient features and thereby improve internal feature-interpretation. Mookkandi et al. [64] present a lightweight vision-transformer architecture (MaxViT) integrated with CBAM (Convolutional Block Attention Module), squeeze-and-excitation (SE) and depth-wise convolutions, and explicitly include a Grad-CAM-based interpretability analysis to visualise which image regions drive predictions. While these works focus on agriculture, they underscore two critical dimensions relevant for multimodal cancer AI systems: (i) module-level architecture explainability (e.g., attention blocks, residual bypasses that allow tracing how information flows) and (ii) visual/rationale explanation of predictions (e.g., attention maps, Grad-CAM saliency) that enable domain-experts to see why a model made a decision. In the context of integrating multi-omics and imaging data for cancer prognosis, such XAI methods—attention-driven architectures, feature attribution, and visual saliency—should be incorporated to bridge the “black-box” gap, enhance clinician trust, support auditability of biomarker-image associations, and facilitate the translation of algorithmic predictions into actionable clinical insights.

### 4.6. Pipeline of Multi-Modal Cancer AI

A general multimodal AI pipeline for cancer research mainly comprises three steps: feature extraction, fusion, and prediction. Imaging features are obtained through deep learning models such as CNNs and Vision Transformers, while omics features—genomics and transcriptomics—are encoded by autoencoders, graph neural networks, or regularized machine learning methods [65]. In medical images, pre-processing often requires segmentation, for which U-Net and nnU-Net [66] are widely used for tumor boundary detection [67]. The strategies of feature fusion can be categorized into three classes: early fusion at the feature level, late fusion at the ensemble level, and hybrid fusion that combines shared and independent representations [68]. Eventually, the prediction be made by classifiers or survival models, followed by calibration and explainability. This pipeline allows one to leverage the complementary information from imaging and omics to improve the prognostic performance and clinical utility.

The pipeline for a multi-modal cancer AI system begins with data acquisition and harmonization, where imaging (e.g., PET/CT), omics (genomics/transcriptomics), and clinical data are pre-processed and aligned. Next, feature extraction and modality-specific encoding transform raw inputs into latent representations. Following this, a fusion layer integrates the modalities—via early, late or hybrid strategies—to learn joint representations. The fused embedding then feeds into a predictive model (e.g., survival, subtype, response). Finally, validation and deployment involve external cohort testing and interpretability analyses to ensure clinical readiness. This structured workflow enables robust, scalable multimodal AI applications in oncology [69].

## 5. Conclusions

Across cancer indications, we observed consistent gains from multimodal learning over single-modality baselines for diagnosis, prognosis, and treatment response prediction. Our synthesis clarified the conceptual taxonomy of early, late, and hybrid fusion; highlighted clinically constrained knowledge-informed models as promising directions; and summarized the modality pairings most frequently investigated (e.g., histopathology-omics and MRI-epigenomics). Clinically, the reviewed evidence suggests clear use cases where multimodal fusion can add value (e.g., non-invasive biomarker surrogates, therapy stratification, and survival prediction).

However, the methodological quality across the reviews varied substantially. Using AMSTAR 2.0, most reviews were classified as critically low or low, mainly due to absence of protocol registration, limited justification of exclusions, and incomplete assessment of bias and publication bias. Only one study reached a moderate confidence rating. These findings do not negate the promise of multimodal AI; rather, they underscore that current conclusions are often based on heterogeneous pipelines, retrospective cohorts, and insufficient external validation. As a result, the translational signal, while encouraging, remains fragile without stronger methodology and standardized reporting. Routine deployment will further depend on closing gaps in generalizability and interpretability, aligning outputs with clinical workflows, and demonstrating decision-level benefit beyond accuracy. In particular, calibration, uncertainty quantification, and decision-curve/net-benefit analyses should complement conventional discrimination metrics. Interoperability with standards (e.g., DICOM for imaging, OMOP/mCODE for clinical/omics) and attention to privacy-preserving learning (e.g., federated pipelines) will facilitate safe and scalable adoption.

As future directions, we recommend (i) protocolized, prospectively registered systematic reviews and living evidence syntheses as the field evolves; (ii) multi-center, prospective and/or pragmatic evaluations that include external validation and pre-specified decision-focused endpoints; (iii) harmonized, reproducible fusion pipelines with common benchmarks, public code, and dataset documentation adhering to FAIR principles; (iv) biologically informed and clinician-in-the-loop hybrid models that balance performance with transparency, including model cards and error analyses; (v) standardized reporting (PRISMA/PROSPERO for evidence syntheses; domain-specific reporting for AI studies) and routine bias/publication-bias evaluations; and (vi) rigorous health-technology assessment, including cost-effectiveness and workflow impact studies. By pairing methodological rigor with clinically grounded objectives, multimodal techniques, in particular hybrid AI and knowledge-based fusion, can transition from promising prototypes to trustworthy tools for precision oncology.

## Figures and Tables

**Table 1:** PICOS Framework for the Umbrella Review on Multiomics and Medical Imaging in AI-based Cancer Research.

Element	Description
Population	Cancer patients
Intervention	AI-based fusion of genomics and imaging
Comparator	None (overview of methods)
Outcome	Accuracy, interpretability, clinical value
Study Design	Systematic reviews or meta-analyses

**Table 2:** Search Strategy for Identifying Relevant Literature.

Search Component	Keywords Used
Study Type (Query 1)	“systematic review” OR “systematic literature review” OR “literature review” OR “meta-analysis”
Omics Data (Query 2)	“multiomics” OR “genomics” OR “transcriptomics” OR “epigenomics” OR “methylation”
Medical Imaging (Query 3)	“imaging” OR “medical imaging” OR “radiomics” OR “MRI” OR “Magnetic Resonance Imaging” OR “CT” OR “Computed Tomography” OR “CT Scan” OR “Computed Tomography Scan” OR “PET” OR “Positron Emission Tomography” OR “histopathology”
Disease Focus (Query 4)	“cancer” OR “oncology”
Integration Approach (Query 5)	“fusion” OR “integration”
Artificial Intelligence Techniques (Query 6)	“AI” OR “artificial intelligence” OR “ML” OR “machine learning” OR “DL” OR “deep learning” OR “transfer learning”
Combined Search (Query-7)	(Query 1) AND (Query 2) AND (Query 3) AND (Query 4) AND (Query 5) AND (Query 6)

**Table 3:** Inclusion and exclusion criteria for the Umbrella Review on AI/ML-based fusion of omics and imaging in cancer research.

Inclusion Criteria	Exclusion Criteria
Systematic reviews or meta-analyses	Original research studies only
Reviews that discuss AI/ML-based fusion of omics and imaging data	Reviews that does not used AI/ML methods on omics and imaging data
Reviews focusing on only omics or only imaging modalities having possibilities of fused together	Reviews on omics and medical imaging modalities on specific topics where fusion is not possible
Studies involving human cancer datasets	Studies involving non-human models or non-cancer conditions

**Table 4:** Summary of Search Results.

Database Name	Count (No. of Article Found)
Scopus	60
PubMed	66
Web of Science (WoS)	Query-1 & Query-2 = 4744 Query-1 & Query-3 = 49,545 Query-1 & Query-6 = 29,382 Query 7 (All) = 45
Dimensions.ai	Query-7 (All) Publications = 72 Datasets = 1 Grants = 2 Patents = 0 Clinical Trials = 1 Policy Documents = 0 Letters = 2 Total = 78
After merging the databases and removing duplicates	51

**Table 5:** Selected Multi-Omics and AI in Cancer for Umbrella Studies.

Study ID	Author(s)	Title	DOI	Year
S1 [27]	Wang, Suixue; Wang, Shuling; Wang, Zhengxia	A survey on multi-omics-based cancer diagnosis using machine learning with the potential application in gastrointestinal cancer	10.3389/fmed.2022.1109365	2023
S2 [28]	Nicora, Giovanna; Vitali, Francesca; Dagliati, Arianna; Geifman, Nophar; Bellazzi, Riccardo	Integrated multi-omics analyses in oncology: a review of machine learning methods and tools	10.3389/fonc.2020.01030	2020
S3 [29]	Osuala, Richard; Kushibar, Kaisar; Garrucho, Lidia; Linardos, Akis; Szafranowska, Zuzanna; Klein, Stefan; Glocker, Ben; Diaz, Oliver; Lekadir, Karim	Data synthesis and adversarial networks: A review and meta-analysis in cancer imaging	10.48550/arXiv.2107.09543	2023
S4 [30]	Jennings, Charlotte; Broad, Andrew; Godson, Lucy; Clarke, Emily; Westhead, David; Treanor, Darren	Machine learning-based multimodal prognostic models integrating pathology images and high-throughput omic data for overall survival prediction in cancer: a systematic review	10.48550/arXiv.2507.16876	2025
S5 [31]	Wysocka, Magdalena; Wysocki, Oskar; Zufferey, Marie; Landers, Dónal; Freitas, André	A systematic review of biologically-informed deep learning models for cancer: fundamental trends for encoding and interpreting oncology data	10.48550/arXiv.2207.00812	2023
S6 [32]	Sartori, Flavio; Codicè, Francesco; Caranzano, Isabella; Rollo, Cesare; Birolo, Giovanni; Fariselli, Piero; Pancotti, Corrado	A Comprehensive Review of Deep Learning Applications with Multi-Omics Data in Cancer Research	10.3390/genes16060648	2025
S7 [33]	Han, Eonyong; Kwon, Hwijun; Jung, Inuk	A review on multi-omics integration for aiding study design of large scale TCGA cancer datasets	10.1186/s12864-025-11925-y	2025
S8 [34]	Chakraborty, Sohini; Sharma, Gaurav; Karmakar, Sricheta; Banerjee, Satarupa	Multi-OMICS approaches in cancer biology: New era in cancer therapy	10.1016/j.bbadis.2024.167120	2024
S9 [35]	Chen, Chongyang; Wang, Jing; Pan, Donghui; Wang, Xinyu; Xu, Yuping; Yan, Junjie; Wang, Lizhen; Yang, Xifei; Yang, Min; Liu, Gong-Ping	Applications of multi-omics analysis in human diseases	10.1002/mco2.315	2023
S10 [36]	Akhoundova, Dilara; Rubin, Mark A.	Clinical application of advanced multi-omics tumor profiling: Shaping precision oncology of the future	10.1016/j.ccell.2022.08.011	2022
S11 [37]	Huang, Sijia; Chaudhary, Kumardeep; Garmire, Lana X.	More Is Better: Recent Progress in Multi-Omics Data Integration Methods	10.3389/fgene.2017.00084	2017
S12 [38]	Dong, Mengmeng; Wang, Liping; Hu, Ning; Rao, Yueli; Wang, Zhen; Zhang, Yu	Integration of multi-omics approaches in exploring intra-tumoral heterogeneity	10.1186/s12935-025-03944-2	2025
S13 [39]	Schneider, Lucas; Laiouar-Pedari, Sara; Kuntz, Sara; Krieghoff-Henning, Eva; Hekler, Achim; Kather, Jakob N.; Gaiser, Timo; Froehling, Stefan; Brinker, Titus J.	Integration of deep learning-based image analysis and genomic data in cancer pathology: A systematic review	10.1016/j.ejca.2021.10.007	2022
S14 [40]	Kirienko, Margarita; Gelardi, Fabrizia; Fiz, Francesco; Bauckneht, Matteo; Ninatti, Gaia; Pini, Cristiano; Briganti, Alberto; et al.	Personalised PET imaging in oncology: an umbrella review of meta-analyses to guide the appropriate radiopharmaceutical choice and indication	10.1007/s00259-024-06882-9	2024
S15 [41]	Prelaj, Arsela; Miskovic, V.; Zanitti, M.; Trovo, F.; Genova, C.; Viscardi, Giuseppe; Rebuzzi, S. E.; et al.	Artificial intelligence for predictive biomarker discovery in immuno-oncology: a systematic review	10.1016/j.annonc.2023.10.125	2024
S16 [42]	Maiorano, Mauro Francesco Pio; Cormio, Gennaro; Loizzi, Vera; Maiorano, Brigida Anna	Artificial Intelligence in Ovarian Cancer: A Systematic Review and Meta-Analysis of Predictive AI Models in Genomics, Radiomics, and Immunotherapy	10.3390/ai6040084	2025
S17 [43]	Doykov, Mladen; Valkanov, Stanislav; Khalid, Usman; Gurung, Jasmin; Kostov, Gancho; Hristov, Bozhidar; Uchikov, Petar; et al.	Artificial Intelligence-Augmented Advancements in the Diagnostic Challenges Within Renal Cell Carcinoma	10.3390/jcm14072272	2025
S18 [44]	Ozaki, Yousaku; Broughton, Phil; Abdollahi, Hamed; Valafar, Homayoun; Blenda, Anna V.	Integrating Omics Data and AI for Cancer Diagnosis and Prognosis	10.3390/cancers16132448	2024
S19 [45]	Restini, Felipe Cicci Farinha; Torfeh, Tarraf; Aouadi, Souha; Hammoud, Rabih; Al-Hammadi, Noora; Starling, Maria Thereza Mansur; Sousa, Cecília Felix Penido Mendes; et al.	AI tool for predicting MGMT methylation in glioblastoma for clinical decision support in resource limited settings	10.1038/s41598-024-78189-6	2024
S20 [46]	Unger, Michaela; Kather, Jakob Nikolas	A systematic analysis of deep learning in genomics and histopathology for precision oncology	10.1186/s12920-024-01796-9	2024
S21 [47]	Mao, Lingchao; Wang, Hairong; Hu, Leland S.; Tran, Nhan L.; Canoll, Peter D.; Swanson, Kristin R.; Li, Jing	Knowledge-Informed Machine Learning for Cancer Diagnosis and Prognosis: A Review	10.1109/TASE.2024.3515839	2024

**Table 6:** Summary of Included Studies on Integrating Multi-Omics and Medical Imaging in AI-Driven Cancer Research.

ID	Cancer Types	Modalities (Omics + Imaging)	Fusion Type	AI Method	Tasks	Main Outcomes	Limitations
S1 [27]	Mixed	Multi-omics	Early	ML (integration)	Dx, Prognosis, Subtyping	Early proof of multi-omics value	Pre-DL era, shallow models
S2 [28]	Mixed oncology	Genomics, Transcriptomics, Proteomics, Methylation	Early, Late, Hybrid	ML tools, pipelines	Prognosis, Biomarkers, Subtyping	Catalog of ML tools for oncology	Tool heterogeneity, limited validation
S3 [29]	Mixed cancers	CT, MRI, PET, WSI pathology (no omics)	Late	GANs, adversarial DL	Data synthesis, Detection	GANs boost imaging analysis	Publication bias, limited clinical use
S4 [30]	Mixed tumors	Multi-omics tumor profiling	Hybrid	Clinical ML pipelines	Precision oncology	Framework for clinical precision medicine	Costly, early-stage
S5 [31]	Mixed	Multi-omics (knowledge-informed encoding)	Hybrid	Biologically-informed DL	Dx, Prog.	Improves interpretability	High computational cost
S6 [32]	Mixed pathology	Genomics + Histopathology	Hybrid	DL (CNNs) + ML	Dx, Prognosis	Pathogenomics fusion improves accuracy	Reproducibility concerns
S7 [33]	Gastrointestinal + mixed	Genomics, Transcriptomics, Epigenomics	Early, Hybrid	ML, DL (survey)	Diagnosis, Subtyping	Multi-omics, single-omics for Dx	Retrospective data, preprocessing heterogeneity
S8 [34]	Mixed diseases	Multi-omics	Early	ML, DL	Disease analysis (Dx, Prog.)	Disease-specific multi-omics patterns	Not cancer-only
S9 [35]	Immuno-oncology	Genomics, Transcriptomics (+ some radiomics)	Hybrid	AI biomarker pipelines	Biomarker discovery	Predictive IO biomarkers found	Risk of bias, endpoint variation
S10 [36]	Mixed (PET)	PET radiomics (umbrella review)	Late	Radiomics + ML	Dx, Staging, Response	PET guides radiotracer choice	PET-only, heterogeneous studies
S11 [37]	Ovarian cancer	Genomics, Radiomics, CT/MRI, Immunotherapy	Hybrid	ML, DL	Dx, Prognosis, Tx response	Strong performance across modalities	Heterogeneous, small cohorts
S12 [38]	RCC	Genomics + CT/MRI	Hybrid	AI, ML	Diagnosis, Risk stratification	AI augments RCC workflows	Limited external validation
S13 [39]	Mixed oncology	Multi-omics + Radiomics/Pathomics	Hybrid	AI, ML	Dx, Prognosis	Fusion > single-modality	Lack of prospective studies
S14 [40]	Glioblastoma	Epigenomics (MGMT methylation) + MRI	Hybrid	ML, DL	Biomarker prediction (MGMT)	Accurate non-invasive MGMT prediction	Bias risks identified
S15 [41]	Mixed	Multi-omics + Clinical + Radiology/Pathology	Hybrid	Knowledge-informed ML	Dx, Prognosis	Improves interpretability	Limited benchmarks
S16 [42]	Mixed	Multi-omics (therapy focus)	Hybrid	ML methods	Therapy stratification	Personalized therapy potential	Harmonization challenges
S17 [43]	Mixed	Histopathology WSI + Omics	Hybrid	ML, DL survival models	Survival prediction	Fusion > unimodal for OS	Preprint, small external validation
S18 [44]	Mixed	Large-scale multi-omics	Early, Hybrid	Deep learning	Classification, Prognosis	Effective across TCGA	No prospective validation
S19 [45]	Mixed	Multi-omics (TCGA datasets)	Hybrid	ML + statistical frameworks	Study design, integration	Provides framework guidance	Not validated clinically
S20 [46]	Mixed	Genomics + Transcriptomics (ITH)	Hybrid	ML integration	Heterogeneity analysis	Fusion captures ITH patterns	Small datasets
S21 [47]	Mixed	Histopathology + Omics	Hybrid	ML, DL	Survival analysis	Multimodal survival	No benchmarks

**Table 7:** AMSTAR 2 Methodological Quality (Critical Domains) for 21 Included Reviews/Meta-Analyses.

ID	Protocol Registered	Search Adequacy	Exclusions Justified	RoB of Included	Meta-Analytic Methods	Publication Bias	Critical Domains Met (0–7)	Overall Confidence
S1	N	PN	N	N	NA	NA	0	Critically low
S2	N	Y	N	N	NA	NA	1	Critically low
S3	NR	Y	PN	Y	NA	NA	3	Low
S4	N	PN	N	N	NA	NA	0	Critically low
S5	N	PN	N	N	NA	NA	0	Critically low
S6	NR	Y	PN	Y	NA	NA	3	Low
S7	N	Y	N	N	NA	NA	1	Critically low
S8	NR	PN	N	N	NA	NA	0	Critically low
S9	NR	Y	PN	PN	NA	NA	2	Low
S10	NR	Y	Y	Y	Y	Y	5	Moderate
S11	NR	Y	PN	Y	NA	NA	3	Low
S12	N	N	N	N	NA	NA	0	Critically low
S13	N	PN	N	N	NA	NA	0	Critically low
S14	N	PN	N	N	PN	PN	1	Critically low
S15	NR	Y	PN	PN	Y	Y	4	Low
S16	N	Y	PN	PN	NA	NA	2	Low
S17	N	PN	N	N	NA	NA	0	Critically low
S18	N	PN	N	N	NA	NA	0	Critically low
S19	NR	PN	N	N	NA	NA	0	Critically low
S20	N	PN	N	N	PN	PN	1	Critically low
S21	NR	Y	PN	Y	NA	NA	3	Low

**Table 8:** Overview of Major Public Datasets for Multi-Omics and Medical Imaging in Cancer Research.

Dataset	Data Modalities	Cancer/Population Coverage	Typical Use (Research Scope)	Access Type
TCGA (The Cancer Genome Atlas) [50]	Genomics, Transcriptomics, Epigenomics, Clinical data	33+ tumor types (11,000+ patients)	Biomarker discovery, Survival analysis, Multi-omics fusion	Open (controlled data for germline variants)
TCIA (The Cancer Imaging Archive) [51]	CT, MRI, PET, Histopathology (WSI)	Linked cohorts to TCGA; multiple disease-specific collections	Radiomics, image-based deep learning, segmentation, multimodal studies with TCGA	Open access (after registration)
CPTAC (Clinical Proteomic Tumor Analysis Consortium) [52]	Proteomics + genomics + transcriptomics + imaging for specific cancers	Breast, colon, ovarian, endometrial, lung, etc.	Proteogenomics; linking omics with imaging and clinical outcomes	Open (some controlled-access biospecimen data)
UK Biobank [53]	MRI, CT, whole-body imaging, genomics, lifestyle/clinical phenotypes	Population-scale cohort (500,000+ participants)	Imaging-genomics association, early disease detection, longitudinal studies	Approved application required
SEER (Surveillance, Epidemiology, and End Results) [54]	Clinical + demographic survival registry	US cancer registry covering >47% of population	Population-level outcomes, epidemiology, survival modeling	Open (controlled limited datasets)
Multi-Institutional Radiomics Repositories (e.g., RIDER, LIDC-IDRI, NSCLC-Radiomics, ACRIN) [55]	CT, PET/CT, radiology images with segmentation labels	Lung cancer, NSCLC, COPD, etc.	Radiomics feature extraction, segmentation benchmarking, multimodal validation	Open access

## Data Availability

The data used to support the findings of this study are available from the corresponding author upon request.

## Supplementary Materials

The following supporting information can be downloaded at: https://www.mdpi.com/article/10.3390/cancers17223638/s1, Table S1: PRISMA 2020 checklist.
